# The differential distribution of bacteria between cancerous and noncancerous ovarian tissues in situ

**DOI:** 10.1186/s13048-019-0603-4

**Published:** 2020-01-18

**Authors:** Qi Wang, Lanbo Zhao, Lu Han, Guoxing Fu, Xiaoqian Tuo, Sijia Ma, Qing Li, Yiran Wang, Dongxin Liang, Miaomiao Tang, Chao Sun, Qing Wang, Qing Song, Qiling Li

**Affiliations:** 1 Department of Obstetrics and Gynecology, First Affiliated Hospital, Xi’an Jiaotong University, Xi’an, Shaanxi China; 2 Department of Gynecological Oncology, Shaanxi Provincial Cancer Hospital, Xi’an, Shaanxi China; 3 Guipei 77, Health Science Center, Xi’an Jiaotong University, Xi’an, Shaanxi China; 4 Omega Bioservices Inc, Norcross, GA USA; 5 Cardiovascular Research Institute, Morehouse School of Medicine, Atlanta, Georgia USA

**Keywords:** Ovarian cancer, Bacteria, 16S rRNA sequencing, Lipopolysaccharide, KEGG

## Abstract

**Background:**

With the improvement of bacterial detection, the theory of the sterile female upper reproductive tract has been frequently challenged in recent years. However, thus far, no researchers have used ovaries as study targets.

**Methods:**

Six women who were diagnosed with ovarian cancer were included in the cancer group, and ten women who were diagnosed with a noncancerous ovarian condition (including three patients with uterine myoma and seven patients with uterine adenomyosis) were included in the control group. Immunohistochemistry staining using an antibacterial lipopolysaccharide (LPS) antibody was used to confirm the presence of bacteria in the ovarian tissues. In addition, 16S rRNA sequencing was used to compare the differences in the bacteria between ovarian cancer tissues and noncancerous ovarian tissues. BugBase and Phylogenetic Investigation of Communities by Reconstruction of Unobserved States (PICRUSt) were used to predict the functional composition of the bacteria.

**Results:**

Bacterial LPS was present in ovarian cancer tissue and noncancerous ovarian tissue, which implied the presence of bacteria in ovarian tissue. When compared to the noncancerous ovarian bacteria at the phylum level, the cancerous ovarian bacteria were composed of increased Aquificae and Planctomycetes and decreased Crenarchaeota. When predicting metagenomes, gene functions associated with the potentially pathogenic and the oxidative stress-tolerant phenotype were enriched in the ovaries of the cancer group. Forty-six significantly different KEGG pathways existed in the ovarian bacteria of the cancer group compared to that of the control group.

**Conclusions:**

Different bacteria compositions were present in cancerous and noncancerous ovarian tissues.

**Trial registration:**

Chines Clinical Trail Registry, CHiCTR1800020018, Registered 11 September 2018, http://www.chictr.org.cn/

## Introduction

Abdominal solid viscera, including the pancreas, kidney, spleen, liver and ovary, have always been believed to be absolutely sterile. However, this concept is being challenged. Leore et al. found that the bacteria in pancreatic tumors could mediate tumor resistance to the chemotherapeutic drug gemcitabine [1]. S. Manfredo Vieira et al. confirmed that *Enterococcus gallinarum* can translocate to the lymph nodes, liver and spleen and drive autoimmunity [2].

The upper female reproductive tract, including the uterus, fallopian tubes and ovaries, has been believed to be absolutely sterile due to the obstacle of the cervix, which is also being challenged. The change in mucins in the cervix during the menstrual cycle may lead to the passage of bacteria [3, 4]. In addition, research has confirmed that the uterus and fallopian tubes represent a functionally united peristaltic pump under the endocrine control of the ovaries [5], which may aid the bacteria to enter the endometrium, fallopian tubes, and ovaries.

With the improvement of bacterial detection, researchers have been investigating the upper reproductive tract. Verstraelen et al. aimed to explore the presence of a uterine bacteria using a barcoded Illumina paired-end sequencing method targeting the V1–2 hypervariable region of the 16S RNA gene [6]. Fang et al. revealed diverse intrauterine bacteria in patients with endometrial polyps using barcoded sequencing [7]. Miles and Chen also investigated the bacteria of the reproductive tract in women undergoing hysterectomy and salpingo-oophorectomy using the 16S RNA gene [4, 8]. However, all the abovementioned researchers used endometrial diseases as their research targets, so the question of whether the ovaries are sterile is still unclear.

In recent years, the bacteria of tumor tissues have become a hot topic for researchers. Aleksandar et al. confirmed that Fusobacterium was enriched in colorectal tumors [9]. In addition, Bullman et al. discovered that the colonization of human colorectal cancers with Fusobacterium is maintained in distal metastases and bacteria stability between paired primary and metastatic tumors [10]. Bacteria-driven or-associated carcinogenesis has been demonstrated not only in CRC but also in the cancers of stomach, lung, prostate, breast, cervix and endometrium [11–15]. However, whether the bacteria in ovarian tissue are associated with ovarian cancer was still a question. Therefore, in this study we compared compositional and functional differences of bacteria in cancerous ovarian tissue and normal ovaries.

In this study, we used immunohistochemistry staining and 16S rRNA sequencing to confirm the presence of bacteria in the ovaries. First, we compared the differences in the ovarian bacteria and its predicted function between cancerous and noncancerous ovarian tissues.

## Material and methods

### Patient characteristics

Sixteen patients were enrolled at the First Affiliated Hospital of Xi’an Jiaotong University. Patients with following criteria were included in cancer group: patient with a preliminary diagnosis of suspected ovarian cancer and undergoing laparotomy, and the pathology was serous ovarian cancer. Patients with following criteria were included in control group: patients with a preliminary diagnosis of uterine myoma or uterine adenomyosis and undergoing hysterectomy and salpingo-oophorectomy. The exclusion criteria were as follows: patients who were pregnant or nursing, patients who used antibiotics within 2 months before surgery, patients who had a fever or elevated inflammatory markers, patients with any types of inflammation, and patients with neoadjuvant chemotherapy.

### Sample collection

Once removed, the ovaries were cut into approximately 1-cm thick ovarian tissue samples using a pair of sterile new tweezers without touching anything else. Then, the collected sample was placed into a sterile tube and placed in liquid nitrogen. Specimens were then transferred to the laboratory and stored at − 80 °C.

### Immunohistochemistry for bacterial lipopolysaccharide (LPS) in ovaries

Immunohistochemistry staining was performed on 5 μm serial sections from routine formalin-fixed, paraffin-embedded (FFPE) tissues. The samples were deparaffinized and rehydrated, and antigen retrieval was performed by microwave treatment for 10 min in EDTA buffer (pH 9.0). Endogenous peroxidase activity was stopped by incubating samples with 0.3% hydrogen peroxide in PBS for 20 min. A DAB substrate kit was used to detect HRP (Zytomed Systems, Berlin, Germany). A ZytoChem Plus HRP Polymer Anti-Rabbit secondary antibody was used according to the manufacturer’s instructions (Zytomed Systems). To find the bacteria, the antibody to LPS core (Hycult Biotech, Uden, Netherlands; Clone WN1 222–5) was used at a concentration of 1:300 overnight at 4 °C.

### 16S rRNA sequencing

DNA extractions were performed by using the Mag-Bind® Pathogen DNA 96 Kit (Omega Biotek, Norcross, USA). DNA was quantified using the QuantiFluor dsDNA System (Promega, Madison, USA). The libraries were prepared using an Illumina 16S Metagenomic Sequencing kit (Illumina, Inc., San Diego, USA) according to the manufacturer’s protocol. The V3-V4 region of the bacterial 16S rRNA gene sequences was amplified using the primer pair containing the gene-specific sequences and Illumina adapter overhang nucleotide sequences. The full-length primer sequences were as follows: 16S Amplicon PCR Forward primer: 5′ TCGTCGGCAGCGTCAGATGTGTATAAGA GACAG-[CCTACGGGNGGCWGCAG] and 16S Amplicon PCR Reverse primer: 5′ GTCTCGTGGGCTCGGAGATGTGTATAAGAGACAG-[GACTACHVGGGTATCTAATCC].

Amplicon polymerase chain reaction (PCR) was performed to amplify the template from the DNA sample input. Briefly, each 25 μL PCR contained 12.5 ng of sample DNA as an input, 12.5 μL of 2x KAPA HiFi HotStart ReadyMix (Kapa Biosystems, Wilmington, USA) and 5 μL of 1 μM of each primer. PCRs were carried out using the following protocol: an initial denaturation step was performed at 95 °C for 3 min followed by 25 cycles of denaturation (95 °C, 30 s), annealing (55 °C, 30 s) and extension (72 °C, 30 s), and a final elongation for 5 min at 72 °C. The reaction mix was removed from the PCR product with Mag-Bind RxnPure Plus magnetic beads (Omega Biotek).

A second index PCR amplification, used to incorporate the barcodes and sequencing adapters into the final PCR product, was performed in 25 μL reactions using the same master mix conditions as described above. The cycling conditions were as follows: 95 °C for 3 min, followed by 8 cycles of 95 °C for 30 min, 55 °C for 30 min and 72 °C for 30 min. A final 5-min elongation step was performed at 72 °C.

The library was checked using an Agilent 2200 TapeStation and quantified using a QuantiFluor dsDNA System (Promega). Libraries were then normalized, pooled and sequenced (2 × 300 bp paired-end read setting) on the MiSeq (Illumina, San Diego, USA) using a 600 cycle V3 standard flowcell producing approximately 100,000 paired-end 2 × 300 base reads (Omega Bioservices, Norcross, USA).

### 16S rRNA sequencing analysis

For each sample, the raw reads were filtered based on sequencing quality using Trimmomatic [16]. The primer and adaptor sequences were removed. Sequence reads with both pair-end qualities lower than 25 were truncated. The software package QIIME was used to perform the 16S rRNA analyses. Sequences were clustered into operational taxonomic units (OTUs) at a 97% similarity cutoff, and the relative abundance was calculated for the OTUs in each sample. All sequences were classified using a native Bayesian classifier trained against the RDP training set (version 9; http://sourceforge.net/projects/rdp-classifier/), and OTUs were assigned a classification based on which taxonomy had the majority consensus of the sequences within a given OTU. The OTUs were then aligned to the Silva database. Alpha diversity (including the Chao 1 index, the ACE index, the Shannon index, the Simpson index and the Evenness index) and the UniFrac-based principal coordinates analysis (PCoA) were performed based on the sample group information.

### The prediction of bacteria function

The relative representation of the bacteria characteristics was predicted using BugBase on the basis of six phenotype categories (Ward et al. unpublished) (https://bugbase.cs.umn.edu/): Gram staining, oxygen tolerance, ability to form biofilms, mobile element content, pathogenicity, and oxidative stress tolerance. This software balances the Kyoto Encyclopedia of Genes and Genomes (KEGG) database, the Integrated Microbial Genomes (IMG4) platform and the Pathosystems Resource Integration Center (PATRIC) system to confirm the contribution of specific OTUs to a community-level phenotype [17–19]. PICRUSt was used to predict the functional composition of a metagenome using marker gene data and a database of reference genomes. Functional differences among the different groups were compared using STAMP software [20, 21].

### Statistics

Analyses were performed in SPSS unless stated above. *P* < 0.05 was considered an indication of statistical significance. The differences in age and parity of patients were assessed with the use of Student’s t-test. The differences in menopausal status, history of hypertension and diabetes were assessed using the chi-square test. Differences in the number of ovarian bacteria taxa were assessed with the use of the Mann-Whitney U test.

## Results

### Participant patients

Sixteen patients who were undergoing oophorectomy or hysterectomy and salpingo-oophorectomy were included in this study. In this study, ten women who were diagnosed with benign endometrial conditions with noncancerous ovaries (including three patients with uterine myoma and seven patients with uterine adenomyosis) were set as the control group, and six women who were diagnosed with ovarian cancer (including two patients who were diagnosed in stage II and four patients who were diagnosed in stage Ш) were set as the cancer group. All diagnoses were based on final surgical pathology after oophorectomy or hysterectomy and salpingo-oophorectomy. Compared with the control group, the age, menopausal status, parity, history of hypertension and history of diabetes in patients diagnosed with ovarian cancer were not significantly different **(**Table 1**)**.

**Table 1:** Clinical characteristics of patients enrolled in the study

	Control group (*n* = 10)	Cancer group (*n* = 6)	*P* value
Age	51.6(45–57)	57.3(46–75)	0.29
Menopausal status			0.12
Pre/Peri	8	2	
Post	2	4	
Parity	5.1(1–13)	3.1(2–5)	0.17
History of hypertension			0.52
Yes	1	2	
NO	9	4	
History of diabetes			0.70
Yes	1	1	
NO	9	5	
Stage (%)			
II		2(33.3)	
III		4(66.7)	
Histotype (%)			
Uterine myoma	3(30)	–	
Uterine adenomyosis	7(70)	–	
Ovarian serous carcinoma	–	6(100)	

The *P*-value of age and parity were assessed by Student’s t-test. The *P*-value of menopausal status, history of hypertension and diabetes were calculated by the chi-square test

### The presence of bacteria in the ovaries

**Fig. 1:**
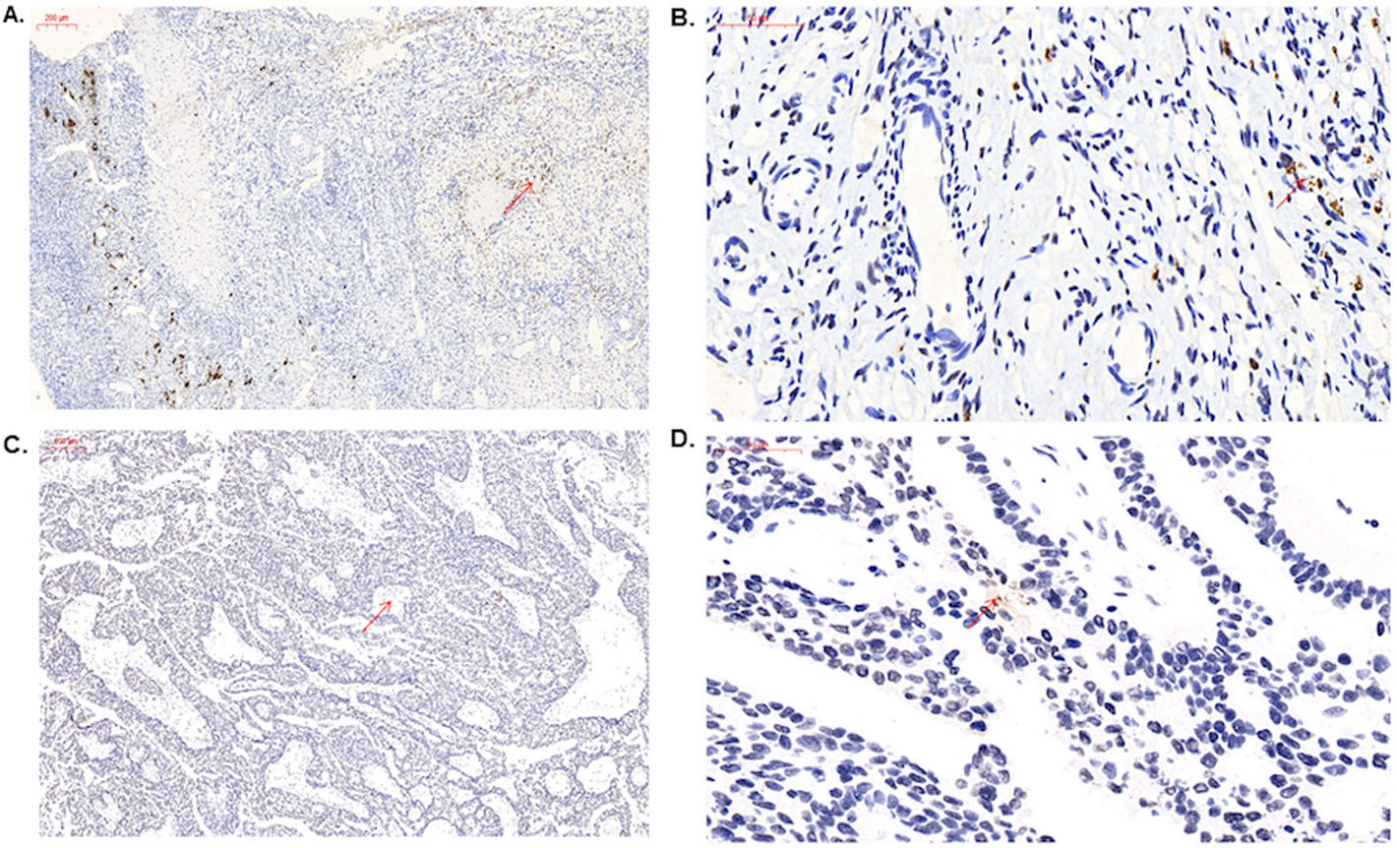
BugBase analysis of predicted metagenomes. The potentially pathogenic and immunohistochemistry of ovaries using an antibacterial LPS antibody. **a** control group (10x). Scale bars, 200 μm. **b** control group (40x). Scale bars, 50 μm. **c** cancer group (10x). Scale bars, 200 μm. **d** cancer group (40x). Scale bars, 50 μm. Arrows point to LPS staining in the ovarian tissue

To confirm the presence of bacteria in ovaries using non-PCR-based methods, we performed immunohistochemistry staining using an antibacterial LPS antibody. The results showed that bacterial LPS were present in the cancerous ovarian tissue and noncancerous ovarian tissue, which implied the presence of bacteria in ovarian tissue **(**Fig. 1**)**.

### Ovarian bacterial richness and diversity between cancer and control groups

**Fig. 2:**
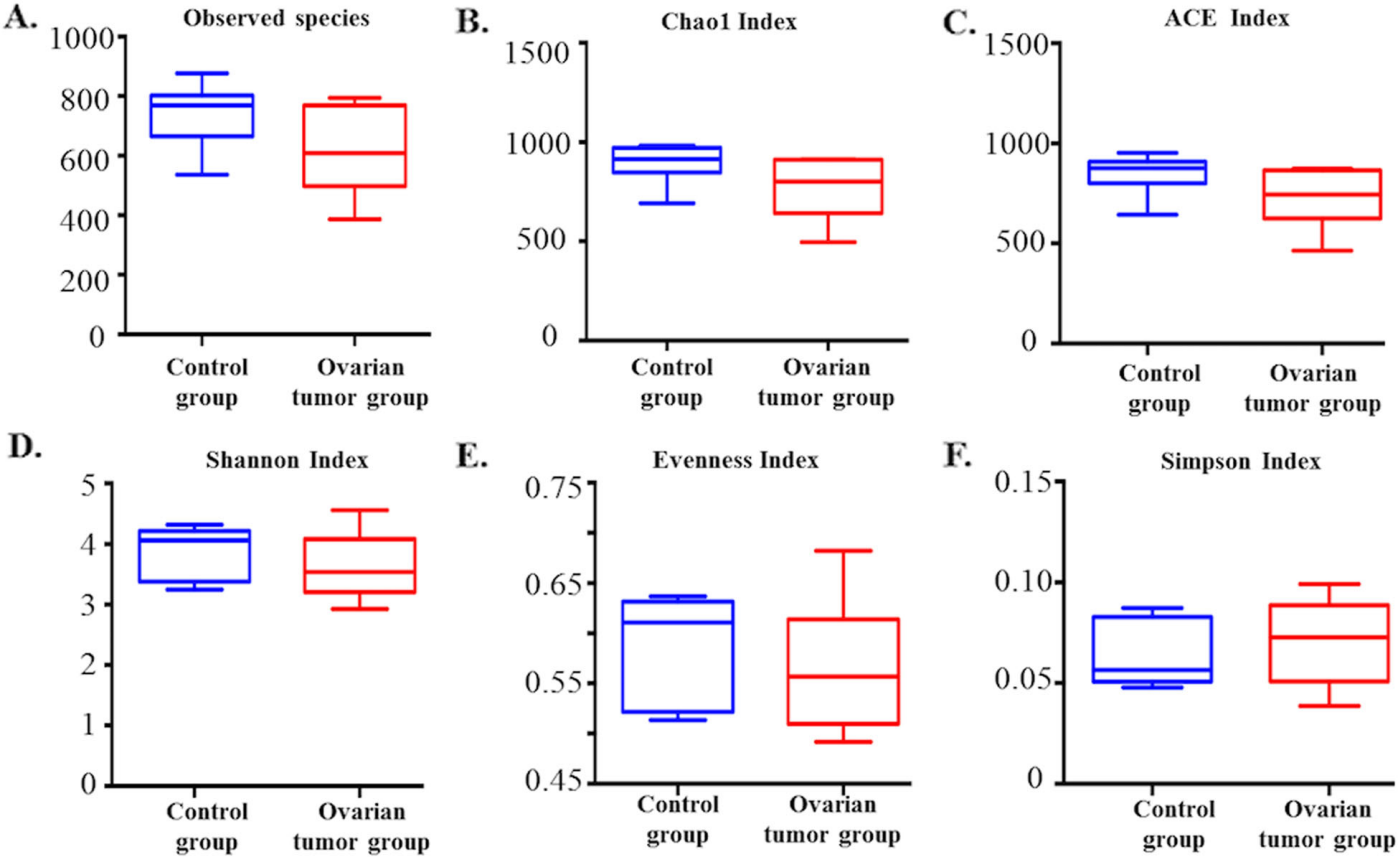
Bacterial richness and diversity in the cancer and control groups revealed by 16S rRNA sequencing **a** Observed species index (*P* = 0.06, Mann-Whitney U test); **b** Chao 1 index (*P* = 0.06, Mann-Whitney U test); **c** ACE index (*P* = 0.06, Mann-Whitney U test); **d** Shannon index (*P* = 0.32, Mann-Whitney U test); **e** Evenness index (*P* = 0.48, Mann-Whitney U test); **f** Simpson index (*P* = 0.46, Mann-Whitney U test)

To detect the ovarian bacterial species richness and diversity between the two groups, we analyzed the alpha diversity of the microbes. The observed number of species in the ovarian cancer tissues was lower than that in the ovaries of the control group, but the difference was not significant. Moreover, we found that not only the bacterial species richness (represented by the Chao 1 index and the ACE index) but also the diversity (represented by the Shannon Index, the Simpson Index, and the Evenness Index) in the ovarian cancer group were not significantly different from those in the control group **(**Fig. 2**)**.

### Ovarian bacteria characterization between cancer and control groups

**Fig. 3:**
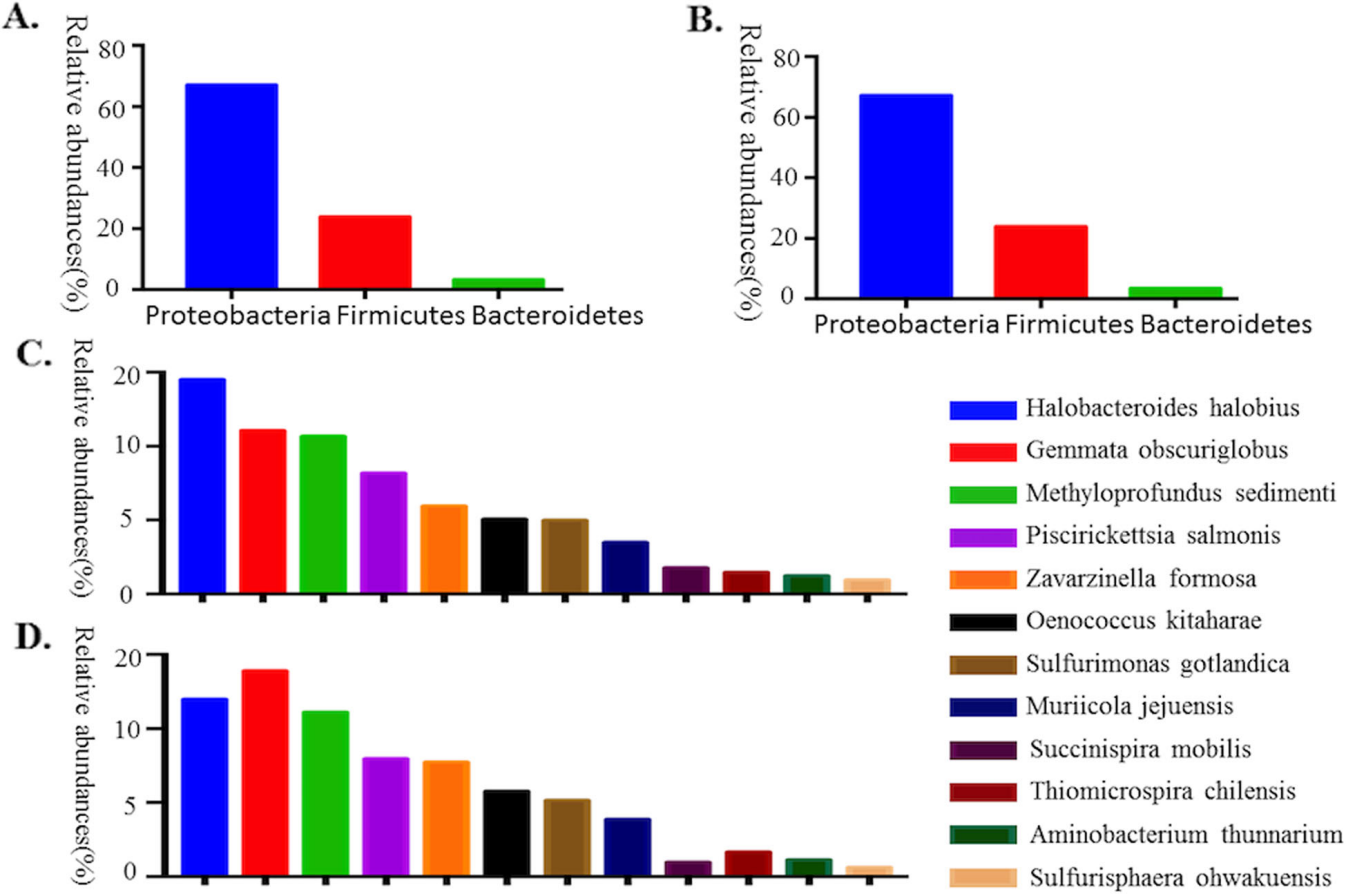
The relative abundance of phyla (> 1%) and the 12 most abundant bacterial species in the ovarian samples. **a** The relative abundance of the phyla (> 1%) in the ovaries of the patients in the control group. **b** The relative abundance of the phyla (> 1%) in the ovaries of patients with ovarian cancer. **c** The relative abundances of the 12 most abundant bacterial species in the ovaries of the control patients. **d** The relative abundances of the 12 most abundant bacterial species in the ovaries of ovarian cancer patients

To understand the ovarian bacteria in cancer and control groups, we performed deep sequencing of the V3-V4 16S rRNA region of all sixteen collected samples. In the ovaries, our results showed that Proteobacteria was the most abundant phylum (67.1% in the control group and 67.20% in the cancer group). Firmicutes was the second most abundant phylum (23.77% in the control group and 23.82% in the cancer group), and the third most abundant phylum was Bacteroidetes (3.26% in the control group and 3.41% in the cancer group) (Fig. 2a, b). At the species level, the ovarian bacterial communities were dominated by *Halobacteroides halobius* (14.53%), followed by *Gemmata obscuriglobus* (11.07%) and *Methyloprofundus sedimenti* (10.69%) in the control group. The ovarian bacterial communities in the cancer group were dominated by *Gemmata obscuriglobus* (13.89%), followed by *Halobacteroides halobius* (11.99%) and *Methyloprofundus sedimenti* (11.12%) **(**Fig. 3**)**.

### Ovarian bacterial community composition differences between cancer and control groups

**Fig. 4:**
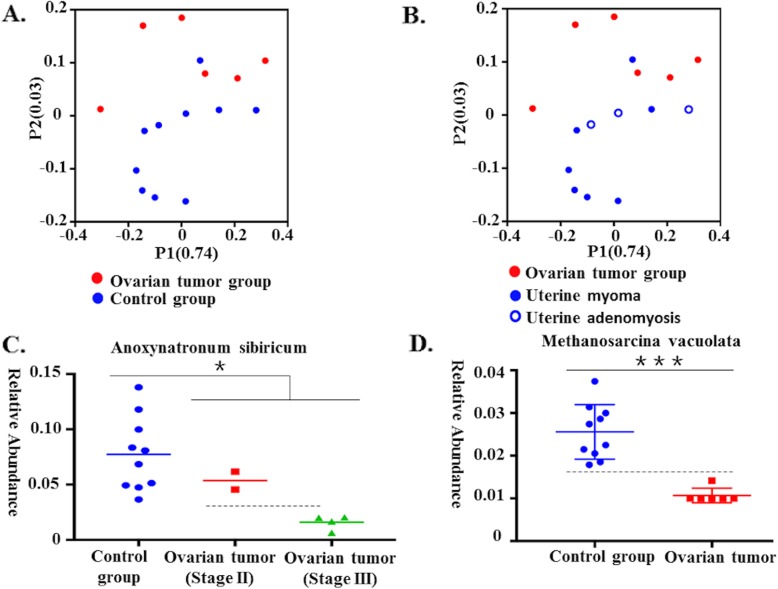
Communities clustered using PCoA and the relative abundance of *Anoxynatronum sibiricum* and *Methanosarcina vacuolata*. **a** Communities were clustered using PCoA. PC1 and PC2 are plotted on the x and y axes. The red block is equal to a sample in the ovarian cancer group. The blue circle is equal to a sample in the control group. The samples from the ovarian cancer group can be separated from other samples in the control group. **b** Communities clustered using Principal Component Analysis (PCoA). PC1 and PC2 are plotted on the x and y axes. The red block is equal to a sample in the ovarian cancer group. The blue solid circle is equal to a sample from a patient with uterine myoma, and the blue hollow circle is equal to a sample of a patient with uterine adenomyosis. **c** The relative abundance of *Anoxynatronum sibiricum* (Control group: *n* = 10, cancer group: *n* = 6, *P* = 0.034, Mann-Whitney U test). **d** The relative abundance of *Methanosarcina vacuolata* (Control group: *n* = 10, cancer group: *n* = 6, *P* = 0.001, Mann-Whitney U test)

We carried out a comparison of differences in the overall bacterial communities using PCoA, which showed that the ovarian bacteria of the control group displayed some differences compared to that of the cancer group **(**Fig. 4a and b**)**.

### Ovarian bacterial composition at different levels in cancer and control groups

To detect the differences in ovarian bacteria between the seventeen samples, we analyzed the ovarian bacterial composition at different levels in cancer and control groups. In the Table 2, we showed the statistical difference of ovarian bacteria in cancer and control groups at phylum, class, order, family, genus and species level. In particular, the relative abundance of *Anoxynatronum sibiricum* may be associated with the stage of the tumor **(**Fig. 4c**),** and *Methanosarcina vacuolata* may be used to diagnose ovarian cancer **(**Fig. 4d**)**.

**Table 2:** Differential relative abundance of the taxa in ovarian communities between patients in cancer and control group

		Control cohort (*n* = 10, %)	Ovarian tumor cohort (*n* = 6, %)	*P* value
Phylum	Planctomycetes	0.5144 ± 0.1420	0.8655 ± 0.2638	0.023
	Crenarchaeota	0.2840 ± 0.0787	0.1592 ± 0.0775	0.023
	Aquificae	0.0352 ± 0.0137	0.0697 ± 0.0291	0.017
Class	Spartobacteria	0.3149 ± 0.0923	0.4795 ± 0.1205	0.026
	Sphingobacteriia	0.1280 ± 0.0695	0.0423 ± 0.0706	0.039
Order	Planctomycetales	7.2700 ± 1.3880	9.1183 ± 0.8594	0.039
	Pseudomonadales	0.1332 ± 0.0746	0.4283 ± 0.4019	0.023
	Enterobacteriales	0.6038 ± 0.1237	2.0105 ± 2.5829	0.030
	Methanobacteriales	0.1626 ± 0.0496	0.2602 ± 0.0859	0.030
	Halobacteriales	0.0648 ± 0.0117	0.0439 ± 0.0287	0.039
	Campylobacterales	0.0776 ± 0.0158	0.1133 ± 0.0232	0.009
Family	Flavobacteriaceae	24.7500 ± 0.6712	21.7167 ± 3.0732	0.014
	Methanobacteriaceae	0.1720 ± 0.0540	0.2667 ± 0.0867	0.039
	Moraxellaceae	0.1328 ± 0.0658	0.4347 ± 0.4054	0.030
	Petrotogaceae	0.0452 ± 0.0178	0.0638 ± 0.0112	0.039
	Thermaceae	0.0078 ± 0.0089	0.0188 ± 0.0086	0.017
	Archaeoglobaceae	0.0611 ± 0.0221	0.0381 ± 0.0123	0.045
	Leptotrichiaceae	0.1018 ± 0.0524	0.0442 ± 0.0284	0.030
	Microbacteriaceae	0.1493 ± 0.0618	0.2740 ± 0.1320	0.039
	Staphylococcaceae	0.0281 ± 0.0545	0.0822 ± 0.0536	0.029
	Thermogemmatisporaceae	0.7381 ± 0.1925	1.4583 ± 0.6982	0.013
	Methanocorpusculaceae	0.0233 ± 0.0139	0.0091 ± 0.0063	0.023
	Geodermatophilaceae	0.0552 ± 0.0335	0.0144 ± 0.0145	0.030
Genus	Paenibacillus	0.7990 ± 0.4563	0.3207 ± 0.2151	0.039
	Haloferula	0.1811 ± 0.0623	0.1156 ± 0.0263	0.023
	Subdivision	0.0801 ± 0.0314	0.0465 ± 0.0188	0.039
	Zavarzinella	0.0741 ± 0.0238	0.1234 ± 0.0305	0.009
	Photorhabdus	0.0013 ± 0.0029	0.0068 ± 0.0050	0.023
	Volucribacter	0.0081 ± 0.0062	0.0021 ± 0.0046	0.042
	Blastococcus	0.0552 ± 0.0335	0.0144 ± 0.0145	0.030
	Mesotoga	0.2509 ± 0.0703	0.3675 ± 0.1057	0.039
	Defluviitoga	0.0550 ± 0.0252	0.0216 ± 0.0114	0.030
	Dorea	0.0063 ± 0.0065	0.0000 ± 0.0000	0.025
Species	Rhodopirellularubra	0.4011 ± 0.1433	0.7563 ± 0.2398	0.013
	Haloferulasargassicola	0.1534 ± 0.0629	0.0999 ± 0.0227	0.030
	Thermogemmatisporafoliorum	0.7813 ± 0.2152	1.4957 ± 0.6735	0.023
	Mycoplasmaequigenitalium	0.5463 ± 0.0684	0.6820 ± 0.1108	0.039
	Bifidobacteriumsubtile	0.0924 ± 0.0269	0.2584 ± 0.1958	0.026
	Natroniellaacetigena	0.0075 ± 0.0078	0.0000 ± 0.0000	0.012
	Flammeovirgakamogawensis	0.6966 ± 0.3523	0.2488 ± 0.1349	0.026
	Eubacteriumyurii	0.0231 ± 0.0111	0.0091 ± 0.0074	0.030
	Enterococcusdiestrammenae	0.2549 ± 0.0859	0.1458 ± 0.0809	0.030
	Pelagicoccusalbus	0.0127 ± 0.0057	0.0047 ± 0.0024	0.017
	Fodinibacterluteus	0.1588 ± 0.0461	0.0935 ± 0.0498	0.039
	Prosthecobacteralgae	0.0210 ± 0.0121	0.0080 ± 0.0050	0.030
	Emticiciaoligotrophica	0.0743 ± 0.0297	0.0308 ± 0.0251	0.013
	Leuconostoccitreum	0.0417 ± 0.0281	0.0108 ± 0.0125	0.039
	Methanimicrococcusblatticola	0.2138 ± 0.0527	0.1572 ± 0.0383	0.039
	Methanosarcinavacuolata	0.0156 ± 0.0061	0.0007 ± 0.0015	0.001
	Lactobacillussucicola	0.0160 ± 0.0063	0.0081 ± 0.0053	0.030
	Caldicoprobacteroshimai	0.0014 ± 0.0041	0.0044 ± 0.0042	0.048
	Caldicellulosiruptorsaccharolyticus	0.3268 ± 0.1880	0.1082 ± 0.1296	0.039
	Methylomicrobiumalbum	0.0013 ± 0.0021	0.0069 ± 0.0051	0.013
	Novispirillum itersonii	0.0031 ± 0.0036	0.0000 ± 0.0000	0.048
	Paenibacillusodorifer	0.6905 ± 0.4128	0.2356 ± 0.1583	0.039
	Mycoplasmagenitalium	0.0023 ± 0.0038	0.0073 ± 0.0048	0.043
	Sulfurospirillumhalorespirans	0.0630 ± 0.0163	0.0948 ± 0.0306	0.039
	Streptococcuscastoreus	0.0514 ± 0.0415	0.0190 ± 0.0329	0.030
	Spongiivirgacitrea	0.2355 ± 0.1391	0.0921 ± 0.0784	0.039
	Staphylococcuscapitissubsp	0.0245 ± 0.0504	0.0752 ± 0.0506	0.021
	Xanthomonasbromi	0.0094 ± 0.0117	0.0000 ± 0.0000	0.025
	Vulcanisaeta thermophila	0.0457 ± 0.0106	0.0720 ± 0.0247	0.039
	Volucribacter amazonae	0.0081 ± 0.0062	0.0021 ± 0.0046	0.042
	Thalassotalea fusca	0.0316 ± 0.0202	0.0027 ± 0.0045	0.004
	Thermus islandicus	0.0051 ± 0.0049	0.0000 ± 0.0000	0.025
	Prevotella veroralis	0.0055 ± 0.0074	0.0000 ± 0.0000	0.048
	Pseudobutyrivibrio xylanivorans	0.0072 ± 0.0063	0.0021 ± 0.0046	0.030
	Peptoniphilus methioninivorax	0.0000 ± 0.0000	0.0031 ± 0.0033	0.017
	Sphingobacterium arenae	0.2488 ± 0.1235	0.0861 ± 0.0529	0.030
	Campylobacter rectus	0.0050 ± 0.0064	0.0000 ± 0.0000	0.048
	Blautia glucerasea	0.0166 ± 0.0091	0.0056 ± 0.0067	0.033
	Calditerricola yamamurae	0.0745 ± 0.0158	0.1084 ± 0.0306	0.023
	Clostridium thermosuccinogenes	0.0036 ± 0.0051	0.0127 ± 0.0089	0.030
	Alkalibacillus haloalkaliphilus	0.0058 ± 0.0066	0.0000 ± 0.0000	0.025
	Acholeplasma oculi	0.0038 ± 0.0041	0.0000 ± 0.0000	0.025
	Aureimonas phyllosphaerae	0.0013 ± 0.0029	0.0068 ± 0.0050	0.023
	Azonexus hydrophilus	0.0773 ± 0.0316	0.0285 ± 0.0190	0.007
	Anaerostipes rhamnosivorans	0.0005 ± 0.0015	0.0045 ± 0.0043	0.025
	Anoxynatronum sibiricum	0.1172 ± 0.0708	0.0460 ± 0.0513	0.034
	Legionella taurinensis	0.0029 ± 0.0031	0.0000 ± 0.0000	0.048
	Mesonia phycicola	0.0119 ± 0.0087	0.0031 ± 0.0033	0.019
	Luteolibacter cuticulihirudinis	0.2389 ± 0.1090	0.4292 ± 0.1517	0.030
	Megasphaera indica	0.0052 ± 0.0055	0.0000 ± 0.0000	0.025
	Dorea formicigenerans	0.0063 ± 0.0065	0.0000 ± 0.0000	0.025
	Fuchsiella alkaliacetigena	0.0082 ± 0.0075	0.0014 ± 0.0031	0.043
	Geobacillus thermodenitrificans	0.0063 ± 0.0051	0.0006 ± 0.0013	0.024

The *P-*value was calculated by the Mann-Whitney U test

### Predicted function of the ovarian bacteria shows phenotypic conservation between cancer and control groups

**Fig. 5:**
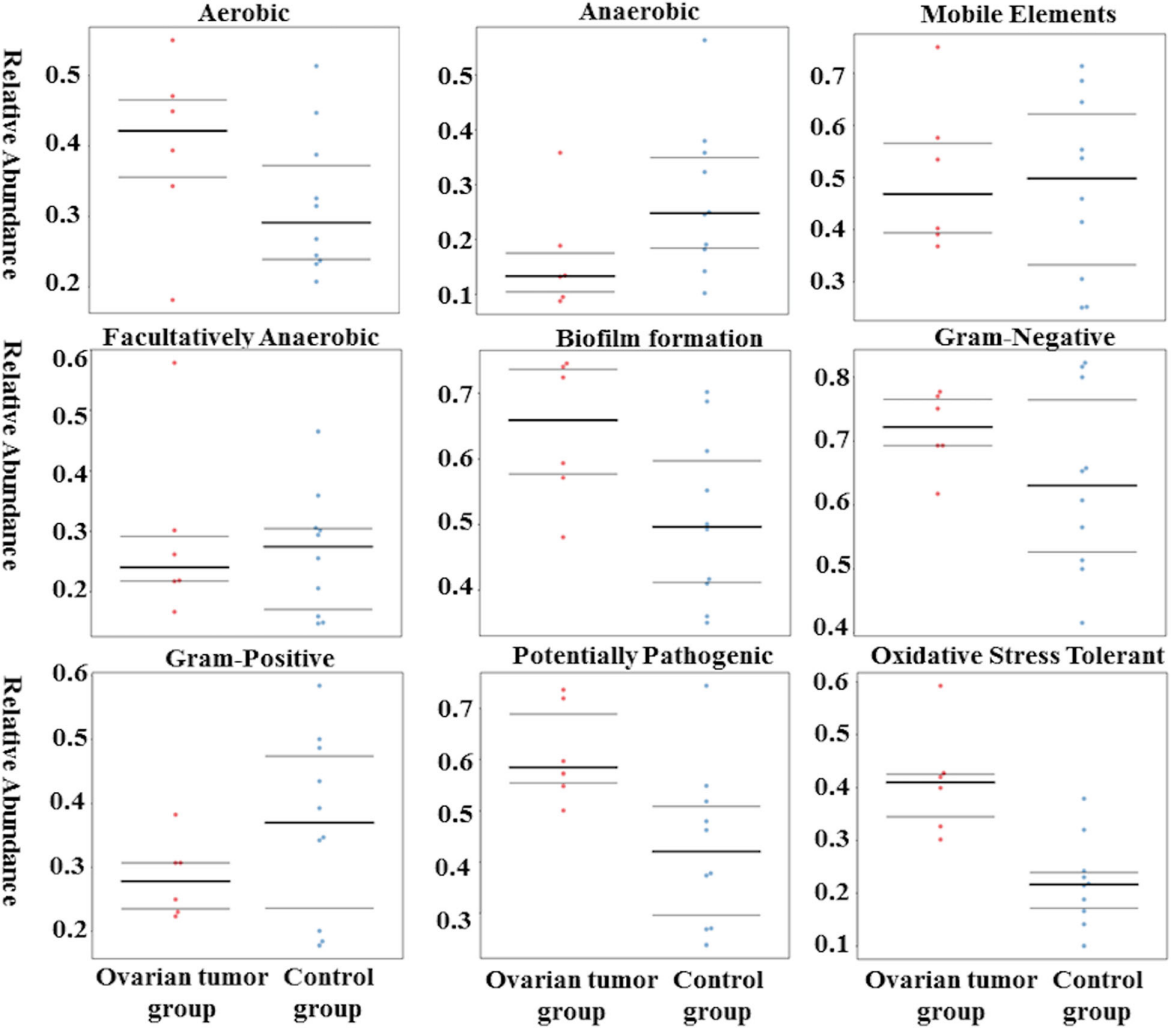
BugBase analysis of predicted metagenomes. The potentially pathogenic and oxidative stress-tolerant phenotype of the ovaries in the cancer group was stronger than that of the control group. (Wilcoxon signed-rank test, *P* = 0.02 and *P* = 0.002)

**Fig. 6:**
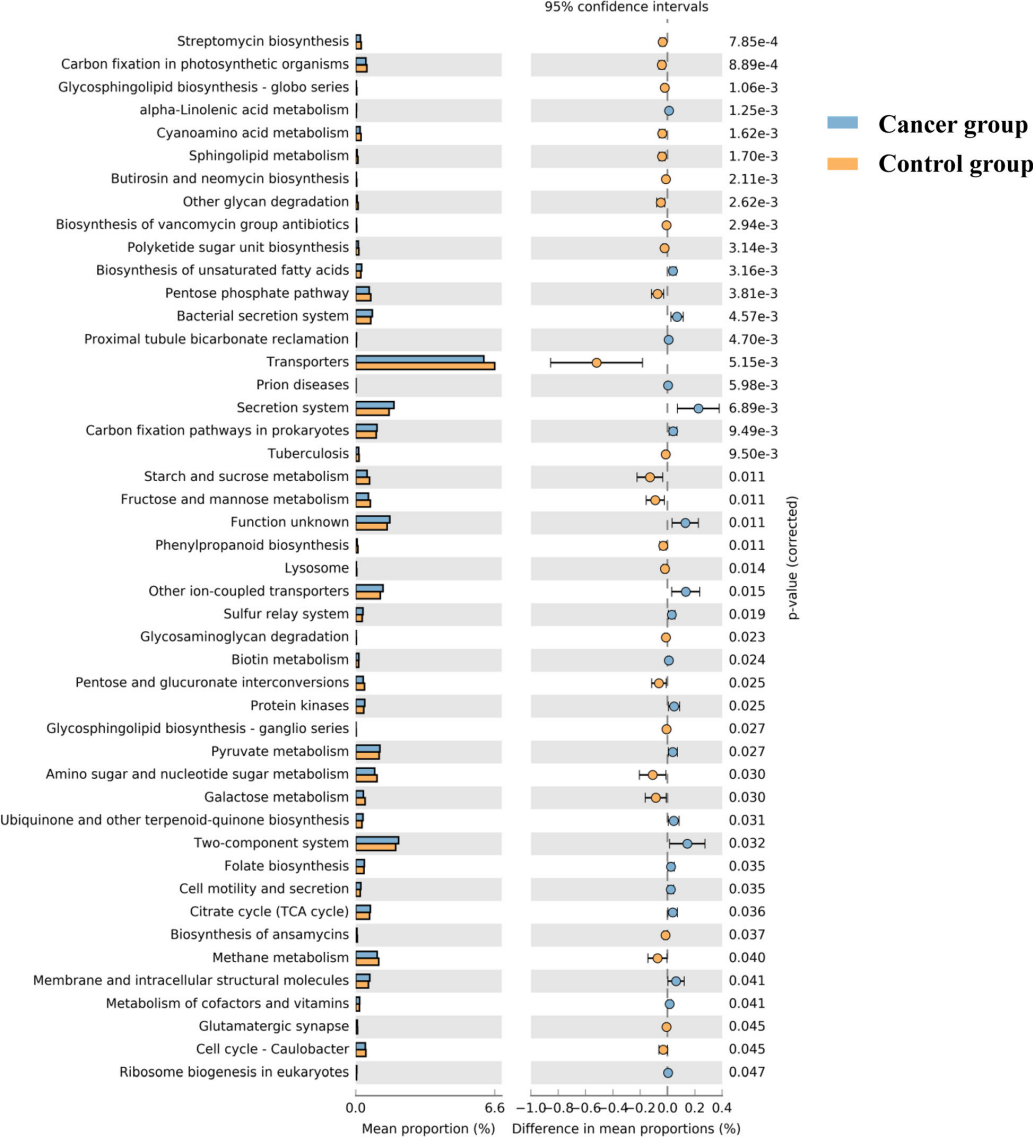
The significantly different KEGG pathways between the cancer and control groups by PICRUSt analysis

BugBase identified that gene functions associated with the potentially pathogenic and the oxidative stress-tolerant phenotype were enriched in the ovaries of the cancer group (Wilcoxon signed-rank test, *P* = 0.02 and *P* = 0.002). The aerobic, anaerobic, facultatively anaerobic, gram-positive, and gram-negative phenotypes; mobile elements; and biofilm formation of the ovarian bacteria showed no significant difference between ovarian cancer and control groups (Fig. 5). PICRUSt was used to identify the KEGG pathways between the bacteria of ovaries in cancer and control groups and found 46 different KEGG pathways. The ovaries in the cancer group showed increased pathways related to streptomycin biosynthesis, carbon fixation in photosynthetic organisms, glycosphingolipid biosynthesis-globo series, cyanoamino acid metabolism, glycerophospholipid metabolism, butirosin and neomycin biosynthesis, other glycan degradation, biosynthesis of vancomycin group antibiotics, polyketide sugar unit biosynthesis, the pentose phosphate pathway, transporters, tuberculosis, starch and sucrose metabolism, fructose and mannose metabolism, phenylalanine metabolism, lysosomes, glycosaminoglycan degradation, pentose and glucuronate interconversions, pyruvate metabolism, amino sugar and nucleotide sugar metabolism, galactose metabolism, biosynthesis of ansamycins, methane metabolism, membrane and intracellular structural molecules, metabolism of cofactors and vitamins, glutamatergic synapse, and the cell cycle. However, the bacteria in ovarian cancer tissue showed reduced alpha-linolenic acid metabolism, biosynthesis of unsaturated fatty acids, bacterial secretion system, proximal tubule bicarbonate reclamation, prion diseases, secretion system, carbon fixation pathways in prokaryotes, unknown functions, other ion-coupled transporters, sulfur metabolism, biotin metabolism, protein kinases, ubiquinone and other terpenoid-quinone biosynthesis, two-component system, folate biosynthesis, cell motility and secretion, citrate cycle (TCA cycle) and ribosome biogenesis in eukaryotes **(**Fig. 6**)**.

## Discussion

Ovarian cancer is the seventh most commonly diagnosed cancer among women that could affect fertility [22]. Most ovarian cancer patients are diagnosed at stages III and IV, and the 5-year survival rate is less than 30% [23]. Researchers have confirmed that the abdominal solid viscera, including the liver, pancreas and spleen, are not absolutely sterile, and the bacteria exist in the upper female reproductive tract as a result of leakage from the cervix [1, 4]. However, the ovaries are still not a research target. The question remains unanswered that whether the ovaries, as one of the abdominal solid viscera, have a bacterium and whether the bacteria has an association with ovarian cancer.

In this study, we first confirmed the presence of bacteria in the ovaries. In addition, we detected significant differences in the ovarian bacteria of patients with ovarian cancer when compared with samples from noncancerous women.

To avoid bacterial contamination, all instruments used were sterilized, and the reagent we used was new. When operating, the surgeon wore an autoclaved mask, cap and suit and did not talk. The sample did not touch anything in the operating room except for the tweezers and was immediately put into the sterilized tube. When the sample was transferred to the laboratory, as many of the procedures as possible were performed on the asepsis work table except the procedures that required large equipment, such as centrifugal machines and sequencers. More importantly, we used ovaries from patients with benign uterine disease as the control group to counteract possible contamination.

There are three possible reasons to explain the origination of the ovarian bacteria. First, a new opinion is that the upper female reproductive tract is not sterile [4], and different bacteria exist throughout the female reproductive tract, forming a continuum from the vagina to the ovaries [23]. The bacteria in the ovaries may originate from the fallopian tubes, uterine cavity, cervix canal or vagina, which is in contact with the outside environment. Besides, many researches have confirmed tubal ligation decreases the risk of EOC by unknown mechanisms [24, 25]. Walther et al. were able to amplify bacterial DNA from 94% of the cervical/vaginal samples and 87% of the uterine samples [23]. However, they were only able to amplify bacterial DNA from 50 and 61% of fallopian tubes and ovaries, which imply the potential mechanisms that tubal ligation impairs circulation of bacteria that are associated with ovarian cancer risk between the lower and upper genital tract [26]. Second, the bacteria in the upper female reproductive tract, including the ovaries, may be endosymbiotic and separated from other bacteria and the outside environment [4]. Third, we put forward a hypothesis that the blood and abdominal cavity may be the potential source of the ovarian bacteria, and its need further exploration [27].

In this study, we found the presence of bacteria in the ovaries and differences in the ovarian bacteria between patients with ovarian cancer and noncancerous women, which raises further questions that need to be solved. Where did the bacteria originate from? What is the association between the bacteria in the ovaries, uterus, fallopian tubes, vagina, and the outside environment? Are the ovarian bacteria always present? The ovaries are connected and open to the abdominal cavity; did the bacteria transfer from the abdominal cavity and the surface of the organs? Moreover, another doubt is whether the ovarian bacteria is associated with ovulation, ovarian failure, ovarian cysts, polycystic ovarian syndrome and so on. Do the ovarian bacteria drive the occurrence of ovarian cancer or does ovarian cancer change the ovarian bacteria? All the above questions point to the direction of our future research.

Our study first concentrates the research target on the bacteria in the cancerous and normal ovarian tissue. The finding about the presence of bacteria in ovarian tissue might start a new field about the association between bacteria and ovarian cancer. Besides, our result about the compositional and functional difference of bacteria in cancerous and normal ovarian tissue might be a new way to explain the carcinogenesis of ovarian cancer and find the therapeutic and prognostic target of bacteria. However, there are some limitations to our study. The first limitation is that we could not collect the ovaries from healthy patients for ethical reasons. Therefore, we used the noncancerous ovaries from patients with benign uterine disease (including uterine myoma and adenomyosis) as the control group. Another limitation of this study is the small sample size, which may limit further analysis and influence the accuracy of the results. However, it is the preliminary study to detect the ovarian bacteria in patients with ovarian cancer, and we will conduct further explorations with larger sample sizes.

## Conclusions

The ovaries contained several kinds of bacteria and were not sterile in a noninflammatory environment. Besides, there were significant differences between the ovarian bacterial compositions of patients in the cancer and control groups.

## Data Availability

Please contact the corresponding author Qiling Li (liqiling@mail.xjtu.edu.cn).

## Abbreviations

FFPE: Formalin-fixed paraffin-embedded
IMG4: Integrated Microbial Genomes
KEGG: Kyoto Encyclopedia of Genes and Genomes
LPS: Lipopolysaccharide
OTUs: Operational taxonomic units
PATRIC: Pathosystems Resource Integration Center
PCoA: Principal coordinates analysis
PCR: Polymerase chain reaction
PICRUSt: Phylogenetic Investigation of Communities by Reconstruction of Unobserved States
